# Ivosidenib for IDH1 Mutant Cholangiocarcinoma: A Narrative Review

**DOI:** 10.7759/cureus.21018

**Published:** 2022-01-07

**Authors:** Vikram Sumbly, Ian Landry, Vincent Rizzo

**Affiliations:** 1 Internal Medicine, Icahn School of Medicine at Mount Sinai, New York City Health and Hospitals | Queens, Jamaica, USA

**Keywords:** oncology, gastrointestinal tumor, precision therapy, ivosidenib, cholangiocarcinoma

## Abstract

Cholangiocarcinoma is an uncommon gastrointestinal neoplasm characterized by the abnormal proliferation of cholangiocytes within the biliary duct. This type of malignancy can be subdivided into three major classes: intrahepatic cholangiocarcinoma (iCCA), perihilar cholangiocarcinoma (pCCA), and distal cholangiocarcinoma (dCCA). Based on the results of various clinical trials, ivosidenib was approved for acute myeloid leukemia harboring the IDH1 mutation. It has also been shown that ivosidenib was effective in patients with IDH1 mutated cholangiocarcinoma. In this article, we briefly review the genomics and prognosis of cholangiocarcinoma with a special focus on ivosidenib and the mechanisms by which its approval was met.

## Introduction and background

Cholangiocarcinoma (CCA) is a malignant neoplasm that arises from the epithelia of the biliary tree, excluding the ampulla of Vater and gallbladder [1]. CCAs are a heterogeneous group of tumors that may be differentiated based on their anatomical location. The three subtypes of CCA - intrahepatic CCA (iCCA), perihilar CCA (pCCA), and distal CCA (dCCA) - differ in anatomical origin, epidemiology, pathogenesis, and management [1].

iCCAs represent approximately 10% of all CCA cases, whereas perihilar and distal disease constitute 50% and 40% of all CCA cases, respectively [2]. Population-based studies revealed that the incidence of CCA in the United States of America (USA) is approximately 11.97 per 1,000,000 person-years [3]. The majority of patients are Caucasian males with a median age of 65 [3]. Worldwide, the rates of CCAs are higher in patients of Hispanic and Asian descent (2.8-3.3 per 100,000) [4].

Although most cases of CCA arise de novo and have no identifiable cause, it has been shown that hepatic cirrhosis, viral agents (e.g hepatitis B and C), ulcerative colitis (UC), primary biliary sclerosing cholangitis (PBSC), and certain hepatic flukes are risk factors [4-5]. The signs and symptoms of CCA are dictated by (1) the tumor location, (2) tumor size, and (3) involved organs [6]. While most patients with iCCA are initially asymptomatic, many may eventually experience generalized malaise, weight loss, fatigue, and abdominal pain [6]. In contrast, biliary obstruction is a common presentation of extrahepatic cholangiocarcinomas (ECCs), which leads to painless jaundice, dark urine, and pale stools [6].

Also known as Tibsovo, ivosidenib is a small molecule IDH1 inhibitor that was approved by the Food Drug Administration (FDA) in 2021 for the treatment of IDH1 mutated locally advanced or metastatic CCA. Ivosidenib was initially approved in 2018 by the FDA for the treatment of acute myeloid leukemia (AML) with an IDH-1 mutation but showed great promise in a recent phase III clinical trial in CCA patients [7]. Based on the results of this trial, ivosidenib was generally well-tolerated by CCA patients and was shown to increase progression-free survival.

## Review

Method

The two first authors conducted the informal search on September 30th, 2021, via the PubMed, ScienceDirect, Google Scholar, and ClinicalTrials search engines. The following keywords and Boolean operators (“AND”, “OR”) were used for the narrative review: “IDH1 mutant cholangiocarcinoma,” “Cholangiocarcinoma,” “Ivosidenib OR Tibsovo,” and “Ivosidenib OR Tibsovo AND Cholangiocarcinoma” (Table 1).

**Table 1:** Number of articles per search engine for specific search terms

Keywords or combination of keywords	PubMed	ScienceDirect	Google Scholar
IDH1 mutant cholangiocarcinoma	59	336	3,620
Cholangiocarcinoma	15,811	25,883	217,000
Ivosidenib OR Tibsovo	137	453	2,540
Ivosidenib OR Tibsovo AND Cholangiocarcinoma	24	451	723

The selected articles were basic science, clinical research, or translational research papers from 2001-2021. Studies were only included if they fulfilled any of the following criteria: (1) Scientific papers written in English from 2001-2021; (2) published in vivo or in vitro studies that elaborated on the genomics of cholangiocarcinomas; (3) published in in vivo or in vitro studies that explained the mechanism of action of ivosidenib and/or the effects of ivosidenib in humans; otherwise, scientific papers were excluded if they met any of the following criteria: (1) case reports or case series; (2) opinion articles or editorials; (3) used data from animals that were non-murine in origin. A total of 36 articles were selected by the first, second, and last authors to create this review article.

Discussion

*Genomics and Biochemical Cascades* Biliary tract cancers (BTCs) are known to harbor various genetic mutations and a high tumor mutational burden (TMB). Cytogenetic abnormalities, such as chromosomal gains (1q, 7p, 8q, 17q and 20q) and chromosomal losses (1p, 3p, 4q, 6q, 8p, 9pq, 13q, 14q, 17p, 18q and 21q), have been observed in different subtypes of cholangiocarcinoma [8-10]. These aberrations promote tumorigenesis by disrupting the normal function of various crucial genes. Nakamura et al. sequenced the genome of 260 patients with BTC (145 iCCAs, 86 pCCAs/dCCAs, and 29 gallbladder cancers) and found 32 significantly altered genes [11]. iCCA cells were more likely to have mutations in BAP1, EPHA2, IDH 1/2, and FGFR 1/2/3, whereas mutations in ARID1B, ELF3, PBRM1, PRKACA, and PRKACB were more predominant in the pCCA/dCCA cells [11-12]. The Cancer Genome Atlas (TCGA) scientists also discovered that gain of function mutations in the TP53 and PTEN oncogenes and loss of functions mutations in the BRAF and KRAS proto-oncogenes contribute to the development of cholangiocarcinoma [13].

According to Sia et al., the mutated genes involved in the pathogenesis of CCA can be classified into two different biological groups: the inflammatory group or the proliferative group [10]. The inflammatory group (62% of cases) is linked to mutations in KRAS and BRAF and is characterized by the abnormal activation of the MET, RAS, and MAPK signaling pathways. These biochemical cascades eventually trigger the RAS-RAF-MEK-ERK signaling axis and PI3K-AKT-mTOR signaling axis, which, in turn, favor cellular proliferation and cellular survival respectively [4,10]. The proliferative group (38% of cases) is associated with the activation of STAT3, overexpression of cytokines, and the activation of various inflammatory pathways [10].

Ong et al. sequenced the genome of eight liver-fluke-associated CCAs and discovered 206 different mutations in 187 genes. The three most altered genes were TP53 (44.4% of all cases), KRAS (16.7%), and SMAD4 (16.7%) [14]. A mutated p53 facilitates the process of oncogenesis by disrupting the process of normal cellular survival, proliferation, death, and DNA repair [15]. Meanwhile, SMAD4 modulates the TGF-β signaling cascade and has been associated with the phenomenon of metastasis [4]. Inactivating mutations in genes such as MLL3 (14.8%), ROBO2 (9.3%), RNF43 (9.3%), and PEG3 (5.6%) have also been shown to contribute to the formation of CCAs by deactivating histone modifiers, activating G protein signaling, and increasing genome instability [14]. *IDH1 and IDH2* Approximately 15%-20% of iCCA patients have mutated IDH 1/2 genes [16]. The isocitrate dehydrogenase 1 (IDH1) and isocitrate dehydrogenase 2 (IDH2) proteins reside in the cytoplasm/peroxisome and mitochondria, respectively. Both obligate homodimers are NADP+-dependent isocitrate dehydrogenases that accelerate the conversion of isocitrate to α-ketoglutarate (α-KG) [17]. iCCA cells with mutations in the IDH 1/2 genes are prone to accumulate the metabolite 2-hydroxyglutarate (2-HG) [18-20]. Tumor cells with wild-type IDH1 and IDH2 were found to contain an average of 3.41 μg of 2-HG per gram of tumor tissue [21]. Borger et al. discovered that 2-HG levels were elevated 248-fold above normal (845.9 μg/g) in IDH1 p.R132C cholangiocarcinoma cells [21]. Samples with the IDH2 p.R172W mutation also had elevated levels of 2-HG (588.5 μg/g) [21]. Therefore, gain-of-function mutations in IDH1 or IDH2 are important triggering events in the development of cholangiocarcinoma.

Normally, 2-HG is produced in very low concentrations and is the byproduct of D-2HG and L-2HG catalysis [22]. Homeostasis is preserved by the conversion of 2-HG into α-KG by chirality-specific dehydrogenases (D-2HGDH and L-2HGDH), but the 2-HG clearance system is rapidly overwhelmed in cells with IDH1 or IDH2 mutations [22]. The excessive molecules of 2-HG then competitively inhibit α-KG-dependent 5-methylcytosine (5mC) hydroxylases (e.g TET2) and histone demethylases (e.g JmjC) [23]. The inhibition of such proteins accelerates the creation of cancerous cells by destabilizing normal epigenetic regulation [24]. Indeed, IDH 1/2 mutant cancers often contain hypermethylated CpG islands in the promoter region of various regulatory genes [25-26]. These silenced genes then impair proper cellular differentiation. EgIN prolyl-4 hydroxylases are enzymes that regulate the degradation of the hypoxia-inducible factor (HIF) transcription factor [26]. These proteins have also been shown to be inhibited by high levels of 2-HG [26]. *Prognosis* David et al. led a retrospective study that included 242 CCA patients and revealed that the median survival is 15.8 months [27]. Those diagnosed with stage I and stage II CCA had a median survival of 23 months and 25 months, respectively, whereas those diagnosed with stage III (14 months) or stage IV (4.5 months) CCA had worse outcomes [27]. Approximately 51% of all stage IV CCAs were iCCA while dCCA (18%) and pCCA (12%) represented 30% of stage IV CCAs [27]. In contrast, pCCA patients were more likely to be diagnosed with stage I disease [27]. Nonetheless, the median survival of unstaged pCCA (17 months) was worse than unstaged iCCA (21.5 months) and dCCA (28 months) [27].

For iCCA patients, the presence of underlying medical conditions (e.g hepatic cirrhosis, diabetes mellitus, inflammatory bowel disease, and primary sclerosing cholangitis) or the act of smoking and drinking alcohol has not been shown to significantly alter the median survival [27]. Surgical resection was performed in 39% of patients as a single modality intervention [27]. Several patients also received a combination of surgical resection with chemotherapy (10%), chemo-radiation therapy (5.5%), or radiation therapy (1.1%) [27]. Other single modality interventions, such as only chemotherapy, chemoradiation, and radiation, were also offered. Surgery was the preferred single modality approach, as it was associated with the highest median survival [27] of 23% when compared to only chemotherapy (8.6%) and only radiotherapy (6.6%). A combination of surgery and chemotherapy achieved the highest median survival in the multimodality group, with 47.3% vs 12.4% in chemoradiation or 13% in surgery with chemoradiation. In contrast, pCCA and dCCA patients who underwent multimodality approaches had significantly better outcomes than those who underwent single interventions [27]. Patients with pCCA who underwent only surgery showed a median overall survival of 18.6% vs 23.4% with surgery and chemotherapy, 33.2% with chemoradiation, and 50% with surgery and chemoradiation. Those patients with dCCA who underwent only surgery had 21.9% median survival with 34.3% in surgery and chemotherapy, 24% in chemoradiation, and 69% overall median survival in surgery with chemoradiation. *Ivosidenib in Clinical Trials* Ivosidenib was initially studied as a single agent in both newly diagnosed and relapsed/refractory AML [28]. In preliminary trials, patients with an IDH1 mutation who were not candidates for intensive chemotherapy were randomized to a starting dose of ivosidenib 500 mg daily and continued therapy until the outcome of disease progression, unacceptable toxicity, or initiation of hematopoietic stem cell transplant. Efficacy was established based on complete remission (Cr) (i.e., less than 5% blasts in the bone marrow, no evidence of disease, and full recovery of peripheral blood counts - platelets >100,000/uL and ANC >1000/uL) or complete remission with partial hematologic recovery (CrPR) (i.e., less than 5% blasts in the bone marrow, no evidence of disease, and partial recovery of peripheral blood counts - platelets >50,000/uL and ANC >500/uL). In patients with newly diagnosed AML, 43% achieved either complete remission or complete remission with partial hematologic recovery. Additionally, 58% of those who achieved remission continued to be in remission at 12 months of follow-up. In relapsed/refractory AML, 33% of patients achieved Cr or CrPR. For patients who achieved remission, the median time to remission was two months and the median duration of the response was 10.1 months (Cr) and 8.2 months (CrPR). Notably, 47% of patients who had received one prior regimen achieved complete remission or complete remission with partial hematologic recovery.

A potentially fatal complication, differentiation syndrome (DS), may result as a product of the inhibition of the mutant enzyme and is characterized by increasing neutrophilia. As the clone differentiates into neutrophils, symptoms such as noncardiogenic pulmonary edema, fever, hypoxemia, pericardial/pleural effusion, and tumor lysis syndrome may occur. Differentiation syndrome may occur as early as Day 1 of treatment and up to three months after initiation of ivosidenib. In the clinical trial, 25% (7/28) of patients with newly diagnosed AML and 19% (34/179) of patients with relapsed/refractory AML experienced differentiation syndrome. Of the seven patients with newly diagnosed AML, six recovered. Of the 34 with relapsed/refractory AML, 27 (79%) recovered after dose interruption [28-29].

Success in IDH1 mutated AML led to the landmark multicenter Phase 3 trial, ClarlDHy [7]. This randomized, double-blind, placebo-controlled trial enrolled patients from 49 different hospitals across six countries. Adult patients with histologically confirmed, advanced IDH1 mutant cholangiocarcinoma, which had progressed while on one or two previous treatment regimens were enrolled and assigned (2:1) to either oral ivosidenib 500 mg or matched placebo. The primary endpoint of the trial was progression-free survival. Over the span of two years, 230 total patients were enrolled in this trial with 185 randomly assigned to either drug vs placebo (124 vs 61) and were then followed at six months. The hazard ratio for progression-free survival was 0.37 (0.25 - 0.54, p<0.001). The median time to progression-free survival was 2.7 months vs 1.4 months in the placebo group [7].

Adverse effects were similar to previously published data, with the most common being fatigue (43%), nausea (41%), abdominal pain/diarrhea (35%), ascites (23%), anemia (18%), and rash (15%). Prolongation of the QT interval and Guillain-Barre syndrome were reported, albeit rare events. Serious adverse effects occurred in 30% of the patients on therapy and 22% of the patients on placebo. There were no treatment-related deaths. The authors concluded that progression-free survival improved with ivosidenib when compared to placebo and was well-tolerated. The major clinical benefit of this trial suggests that targeting IDH1 mutations in advanced cholangiocarcinoma can have a significant benefit on survival [7].

Other Potential Candidates

A lack of effective treatment options is a clinical driver for novel therapies. While the results of ivosidenib in the treatment of advanced cholangiocarcinoma show promising results in progression-free survival, the highly mutagenic nature of these aggressive malignancies warrants the discovery of multiple inhibitors within the pathway for disease progression. As approximately one-fifth of intrahepatic cholangiocarcinoma are thought to harbor IDH1/2 mutations, the pursuit of multiple methods of inhibition is of great importance. The production of the oncometabolite D-2HG leads to metabolic vulnerability by certain known and trusted therapies; chiefly, metformin and chloroquine [30-31]. Previous studies show that metformin and chloroquine increase metabolic stress in mutated cells by selectively inhibiting electron transport chain complex I and increasing the autophagy of mutated cells [32]. Molenaar et al. performed a dose-finding study in patients treated with combination metformin and chloroquine and hypothesized that this combination could be a safe and effective anticancer agent and warrants further exploration as a standalone therapy for patients with IDH mutated cancers [32].

Meanwhile, a phase II trial currently underway at the National Cancer Institute is assessing the effectiveness of the PARP inhibitor olaparib in the treatment of patients with glioma, cholangiocarcinoma, or other solid tumors with IDH1 or IDH2 mutations. The primary endpoint of this trial is the overall response rate in subjects within the three tumor cohorts, with secondary goals of survival assessment, duration of response, and safety of olaparib as monotherapy [33].

Potent, selective, covalent inhibitors of IDH-1 (LY3410738) are also being studied in advanced cholangiocarcinoma by Pauff et al. [34]. This phase I study with a dose-escalation cohort has a primary objective of determining the maximum tolerated dose when administered alone or with the current standard of care. The authors state that this inhibitor binds uniquely to the mutant enzyme, increasing its potency and enabling activity in second-site mutation points.

Olutasidenib (FT-2102) is an IDH-1m inhibitor that has been shown to have a clinical response with mutation clearance in patients with acute myeloid leukemia [35]. A phase I/II study is currently underway assessing five separate cohorts of IDH-mutated tumors, including glioma, hepatobiliary tumors, chondrosarcoma, intrahepatic cholangiocarcinoma, and other solid tumors [36]. Part 1 will attempt to assess dose confirmation followed by the investigation of clinical activity within each cohort. Part 2 will combine olutasidenib with other anticancer agents such as azacitidine, nivolumab, and current standard of care gemcitabine plus cisplatin.

Limitation

The articles used for this review were extracted from four different search engines. It is entirely possible that we missed important articles only available on other search engines. Furthermore, due to the genomic complexity of CCAs, we only briefly reviewed several mutant genes. A broader understanding of all the mutated genes could give the reader a better understanding of CCA pathogenesis. In addition, a more detailed analysis of CCA genomics could lead to the development of future drug targets.

## Conclusions

CCA, also known as bile duct cancer, is a gastrointestinal tumor that originates from the epithelium of the biliary tract. This malignancy can be caused by viral hepatitis, inflammatory bowel disease, or parasitic infections, but mainly arises de novo and often bears a terrible prognosis. Although the exact pathogenesis of CCA remains poorly understood, it has also been linked to mutations in KRAS, TP53, and IDH1. Ivosidenib is a first-in-class IDH1 inhibitor that has been approved by the FDA for the treatment of advanced or metastatic IDH1 mutated CCA. A better understanding of mutated genes and disrupted biochemical pathways may lead to the development of more targeted therapies. This could potentially reduce disease incidence and prolong survival.
